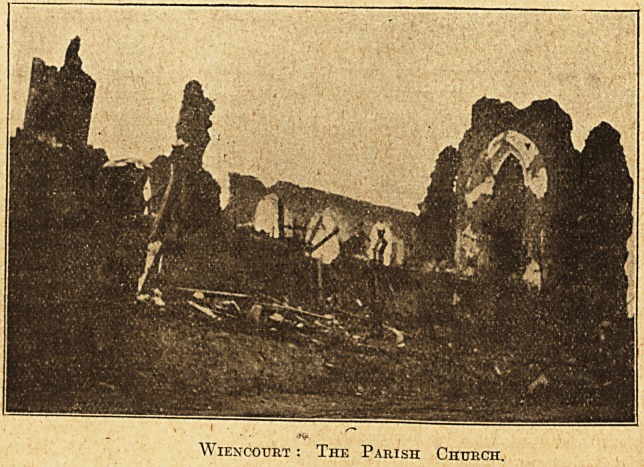# The Army Chaplain

**Published:** 1919-04-26

**Authors:** A. Lombardini


### THE ARMY CHAPLAIN.

By A. LOMBARDINI, C.F.

The "Padré" is essentially a product of the war. The Army Chaplains numbered 113 in August 1914, and were representative of the Church of England and the principal Nonconformist bodies. The "Padré," as he is known to the troops to-day, was evolved when the Territorials crossed the Channel. When the appeal for volunteers was made, an increase in the number of Chaplains was imperative. Facilities were granted by the ecclesiastical authorities to enable men to leave their parishes, with the result that the War Office was inundated with offers of service from deanery, parsonage, and clergyhouse. The Chaplain-General, with his wide knowledge of men and their needs, made his selection, and, after medical examination, drafts of clergymen were despatched to the various schools of instruction, to pass through courses and tests in gas, riding, cooking, drill, and every subject which might serve to make them efficient in their work. May not the work of an Army Chaplain fitly be compared with that of the institutional chaplain? The "padré" ranks as a captain, and receives the honour due to that rank. The wearing of the three stars is a mixed blessing. It gives him the advantage of personal intercourse with the officers and the enjoyment of certain other privileges, but it tends to create a barrier between himself and the men, for whose sake mainly he has given up his work.

Soldiers are students of character, and exceedingly critical, especially of the "padré," and in a very short space of time their approval or disapproval is expressed: either he is "No bon!" or the "Right stuff."

In the early days of the war the Tommy was shy of the "padré"; he did not understand him or the reason why he had joined up. His argument was that in the midst of war there was no time for sermonising, and the kind of men who had been taking Sunday-school services and mothers' meetings would be useless in the field. Four years of war has altered this, and the suggestion that ordination implies loss of manliness has been proved absurd. The work of the "padré" is never at an end; his scope is unlimited. He gets to know and love his men, and for their sake he is out to serve, whatever be the nature of that service. It was not because a certain "padré" could play the tin whistle that the men smiled when they met him in the rest camp, but because of the memory that the shrill and jerky air made them oblivious of the length of the march and the soreness of their feet It was not only because they wanted tobacco that they welcomed him when he came along the trench, but because they knew the enemy were heavily shelling the road along which he had come. And if things were going wrong at home, and a letter had to be written, they knew whom to ask for advice and help. When the time came "to get up and over, and keep behind the barrage," it was the "padré" who said, "Good luck, boys, and God bless you"; and many a man recognised the same voice again which said, "Stick it out, old man," as a hand tore the bandage from the tunic and fixed it on while the bullets splashed in the mud.

Wiencourt: The Parish Church.

The devotion of the "padrés" of the English Church has won for them three V.C.s, ten C.M.G.s, thirty-seven D.S.O.s, 205 M.C.s and bars, five foreign decorations, and two V.D.s. The three V.C.s were won by the Revs. E. N. Mellish, W. R. F. Addison, and T. B. Hardy (who has since died of wounds). But over and above these well-deserved rewards, the "padrés" have won the love and admiration of thousands. In war the winning of a heart is a necessary step in the salvation of a soul. Tommy never wants his "padré" to forget he is a parson, and amongst his happy memories of the war there will always be those of talks around the fire, and the singing of the well-known hymns which linked him with home. The opportunities of a "padré" never cease. It may be that he can carry some one's pack on a march, or go ahead and find billets for the night, and a last "drop in" to say "Good-night"—well, it just makes all the difference, and men understand a religion of this kind. Some have attempted to describe it, and say it is "The Gospel in action." For the "padré" it is the opportunity of his life, for he sees men at their very best. The spirit of sacrifice, bravery, and cheerfulness is revealed, and the opportunity of sharing in a common danger binds one and all together in a comradeship which makes farewell a genuine grief.

Very little is heard of sects or denominations in the Army. A spirit of mutual respect and admiration has manifested itself amongst the ministers of all the Churches, and if the lessons learnt in war can be developed in peace, a happy and useful progress will have been accomplished. But the influence of the "padré" has not been limited to the war zone. There have been thousands of homes in England which have been consoled by a notification other than that which was "official." The complete number of the men who have died of wounds is yet to be published. The majority of those men had a personal message for some one, and it was to the "padré" that the delivery was entrusted. When he writes home and tells the friends or relations

**The Army Chaplain**—(*continued*).

of the military honours at the funeral, and where they will find the grave, they treasure the letter as an heirloom, and ask for further communication in which the details can be amplified.

At the beginning of this year there were 3,463 Church of England Chaplains holding commissions, 1,695 of whom were in France. The number of clergy who have offered their services during the war is 7,169; the number of killed and wounded 264.

Although the war is over, there is a great field open for the work of the Chaplains' Department. Men are being selected for educational purposes, and schools of instruction are being formed in every centre where troops are stationed. From the ranks of the British Army hundreds of men have expressed their desire to become "padres" themselves, and for them special arrangements are being made. A test school has been organised, and very soon many of them will be theological students in the College at Knutsford, with their "padrés" as their tutors.

## Figures and Tables

**Figure f1:**